# Sevoflurane pretreatment alleviates hypoxia-reoxygenation-induced myocardial cell injury by upregulating miR-21-5p

**DOI:** 10.3389/fcvm.2025.1515160

**Published:** 2025-04-01

**Authors:** Saiwen Qiu, Hui Chen, Qifang Jiang

**Affiliations:** ^1^ Department of Anesthesiology, Lanxi Traditional Chinese Medicine Hospital, Lanxi, Zhejiang, China; ^2^ Department of Anesthesiology, Lanxi People’s Hospital, Lanxi, Zhejiang, China

**Keywords:** sevoflurane, miR-21-5p, myocardial protection, hypoxia/reoxygenation, ischemia reperfusion

## Abstract

**Background:**

This study investigates the preventive benefits of sevoflurane against myocardial ischemia-reperfusion (I/R) injury, focusing on its effect on the modulation of miR-21-5p.

**Methods:**

In the clinical study, patients with a history of myocardial ischemia or other conditions requiring surgery were enrolled. Before surgery, the patients were anesthetized with either sevoflurane or propofol. The expression levels of IMA, H-FABP, IL-1β, TNF-α, and IL-6 were also examined. Additionally, the expression of miR-21-5p and its relationships with IMA and H-FABP. A cardiomyocyte hypoxia/reoxygenation (H/R) cell model was created for the *in vitro* tests. The cells were treated with or without sevoflurane and then transfected with inhibitors of miR-21-5p or a negative control (NC). Evaluations were conducted on cell viability, apoptosis ratio, and oxidative stress indicators (MDA, SOD, and ROS). Furthermore, the expression levels of miR-21-5p, apoptotic markers (BCL-2, BAX), myocardial damage markers (IMA, H-FABP), and inflammatory agents (TNF-α, IL-1β, IL-6) were quantified.

**Results:**

In patients with a history of myocardial ischemia, sevoflurane reduced myocardial I/R injury. These patients also showed upregulation of miR-21-5p, which expression positively linked with levels of IMA. Moreover, in H/R treated cardiac cells, sevoflurane markedly reduced the expression of BAX, MDA, ROS, SOD, inflammatory factor and the apoptotic ratio. Nevertheless, inhibition of miR-21-5p abolished these protective effects. Furthermore, in H/R myocardial cells, sevoflurane increased BCL-2 expression and cell survival; these effects were also countered by blocking miR-21-5p.

**Conclusion:**

Mechanistically, we demonstrate for the first time that sevoflurane alleviates myocardial cell injury in myocardial I/R by upregulating miR-21-5p, thereby reducing inflammation, apoptosis, and oxidative stress in myocardial cells. This finding provides a potential therapeutic target for improving myocardial I/R.

## Introduction

Elderly people's lives and health are seriously threatened by cardiovascular disease (1). Acute myocardial infarction remains a life-threatening issue in cardiovascular diseases, often leading to ischemic heart failure. However, myocardial I/R damage cannot be entirely prevented during treatment (2). The term “myocardial ischemia/reperfusion” describes the process of bringing blood back to the heart tissue following an interval of oxygen scarcity (3). Although restoration of blood flow is crucial for preserving ischemic tissue, the reperfusion process itself can cause further myocardial damage (4). Moreover, because of its intricate mechanisms, myocardial I/R injury frequently results in tissue damage and systemic inflammatory reactions, which complicate patient treatment (5). Thus, reducing I/R injury has become a crucial issue in medical research.

Sevoflurane, a fluorinated derivative of methyl isopropyl ether, is an inhalation anesthetic widely used in clinical anesthesia due to its rapid onset and easy control, with a blood-gas partition coefficient of 0.69 (6, 7). Sevoflurane preconditioning has been proven effective in reducing I/R injury. Recent studies indicate that sevoflurane can inhibit inflammatory responses and apoptosis in myocardial I/R (8). Although clinical and experimental evidence demonstrated the cardioprotective effects of sevoflurane, making it an effective drug for reducing the severity of I/R injury during treatment (9, 10). The potential mechanisms by which sevoflurane mitigates myocardial I/R damage remain unclear and warrant investigation.

More and more research is points to ncRNAs as significant regulators of a number of physiological processes (11, 12). Research findings suggest that miRNAs, being potent modulators of gene expression, are intimately linked to a range of cardiovascular conditions, significantly contributing to the onset and progression of both acute and chronic cardiovascular disorders (13, 14). A growing body of evidence has linked several miRNAs was linked to myocardial I/R damage in recent years (15). And miR-21-5p participates in several physiological processes and protects the heart during myocardial infarction (16). Additionally, miR-21-5p can protect myocardial cells from I/R-induced apoptosis (17). According to recent research, miR-21-5p is crucial for fostering heart repair and lowering inflammatory reactions (18), significantly lowering mitochondrial fission and apoptosis caused by I/R, therefore minimizing I/R damage to the heart (19). Furthermore, research has shown that of miR-21-5p is upregulated in sevoflurane preconditioning of spinal cord I/R (20). However, whether sevoflurane preconditioning mitigates myocardial I/R damage by regulating miR-21-5p expression remains unclear.

This study focuses on exploring the alleviative effects of sevoflurane on myocardial I/R injury through the regulation of miR-21-5p, primarily through pathways involving inflammation, oxidative stress, and apoptosis, and further evaluates its impact on myocardial function recovery.

## Methods

### Study subjects

A total of 89 patients who underwent abdominal surgery at our hospital from March 2022 to March 2024 were included in the study, with ages ranging from 40 to 70 years. All participants provided informed consent. This study only selected patient blood samples that met specific criteria, all of which were obtained from patients who provided informed consent. The selection criteria included the patient's disease status and relevant clinical information. To minimize potential selection bias, we strictly adhered to the predefined criteria for sample selection, ensuring that the characteristics between groups were as similar as possible.

Inclusion Criteria: (1) According to the 2014 ACC/AHA/AATS/PCNA/SCAI/STS guidelines for stable ischemic heart disease, the patient's cardiac risk level was classified as ASA II to III (21). (2) Patients undergoing abdominal surgery. (3) Patients without a history of myocardial ischemia, including no history of myocardial ischemia or cardiovascular diseases. (4) Patients with a history of myocardial ischemia, including conditions such as angina, myocardial infarction, and coronary heart disease, who had undergone relevant examinations before surgery confirming the presence of myocardial ischemia. Exclusion Criteria: (1) Patients with severe organ dysfunction, such as severe liver or kidney failure, or dysfunction of other vital organs. (2) Patients with cardiovascular diseases other than myocardial ischemia, such as heart failure, severe hypertension, or arrhythmia, especially those with serious symptoms or complications. (3) Patients with acute or chronic infectious diseases, such as active tuberculosis, hepatitis, or other infections, or those who have been on long-term immunosuppressants or steroids that might affect inflammation or the immune system, will be excluded. (4) Pregnant or breastfeeding women to ensure safety in the study. (5) Patients with other serious diseases such as cancer or blood disorders that might affect the treatment or research intervention. To better investigate the role of sevoflurane in myocardial I/R injury, we divided the blood samples into three groups based on the anesthetic used during surgery and the presence or absence of myocardial ischemia: non-ischemic + sevoflurane group (28 cases), ischemic + sevoflurane group (30 cases), and ischemic + propofol group (31 cases). The propofol group was selected as the control due to its widespread clinical use and its known suppressive effects on the cardiovascular system, making it an appropriate comparator for studying the effects of sevoflurane on myocardial ischemia-reperfusion injury. Additionally, blood samples from patients without a history of myocardial ischemia were chosen as a control, as they more clearly reflect the differences in myocardial injury markers, inflammatory factors, and miR-21-5p expression in patients with a history of myocardial ischemia. The study was approved by the Ethics Committee of Lanxi Traditional Chinese Medicine Hospital.

#### Anesthesia method and intraoperative hemodynamics

Anesthesia was maintained with a mixture of 1.5%–2.0% sevoflurane (C4H3F7O, 95.5%, 28523-86-6, Jiangsu Hengrui Medicine Co., Ltd., Lianyungang, China) and oxygen via inhalation. The sevoflurane concentration was maintained at 2.0%–2.3% in the alveoli to achieve the anesthetic effect. Inhalation of the anesthetic was stopped during skin suturing, and the patient was then transferred to the anesthesia recovery room postoperatively. Propofol (A18293, Adooq Bioscience, Irvine, California, USA) was intravenously infused at a continuous rate of 50–200 *μ*g/kg/min. A blood sample was collected for analysis 24 h after the surgery. Intraoperatively, hemodynamic changes were closely monitored. Blood pressure was maintained within the range of 90–120 mmHg for systolic pressure and 60–80 mmHg for diastolic pressure, with a mean arterial pressure (MAP) of 70–100 mmHg. Heart rate was kept between 50 and 100 beats per minute, and oxygen saturation was maintained between 95%–100%. Respiratory rate was kept at 8–15 breaths per minute, with end-tidal carbon dioxide (EtCO_2_) maintained at 35–45 mmHg. Body temperature was kept within the range of 36.5 °C–37.5 °C. Blood gas analysis was monitored to maintain the pH between 7.35–7.45, PaCO_2_ between 35 and 45 mmHg, and PaO_2_ between 80 and 100 mmHg. Blood glucose levels were controlled between 70 and 120 mg/dl, and hemoglobin concentration was maintained between 12 and 18 g/dl for males and 11–16 g/dl for females. These parameters reflect the physiological stability of the body under anesthesia. Any abnormalities should prompt timely adjustments to the anesthetic protocol or appropriate interventions.

Here is the translation:

Evaluation Indicators

Demographic and Clinical Characteristics: Gender, age, education level, surgery time and type, comorbidities, ASA classification. Demographic and clinical characteristics are shown in Table 1.

**Table 1:** Demographic and clinical characteristics.

Characteristic	Myocardial ischemia + sevoflurane group (*n* = 30)	Myocardial ischemia + propofol group (*n* = 31)	Non-myocardial + sevoflurane group (*n* = 28)
Gender (cases)	Male: 16	Male: 15	Male: 14
	Female: 14	Female: 16	Female: 14
Age (years)	56.5 ± 6.66 (43–69)	55.45 ± 6.94 (43–70)	52.57 ± 7.85 (40–69)
Education Level (individuals)	Primary school: 4	Primary school: 3	Primary school: 2
	Junior high school: 7	Junior high school: 4	Junior high school: 7
	High school: 15	High school: 11	High school: 13
	Junior college: 3	Junior college: 4	Junior college: 2
	Undergraduate: 6	Undergraduate: 8	Undergraduate: 5
	Graduate students: 2	Graduate students: 2	Graduate students: 3
Type of surgery (cases)	Cholecystectomy: 14	Cholecystectomy: 16	Cholecystectomy: 14
	Pancreatectomy: 16	Pancreatectomy: 15	Pancreatectomy: 14
Duration of surgery/minute (minutes)	106.5 ± 49.81 (85–240)	103.8 ± 45.58 (90–240)	101.79 ± 47.45 (90–240)
Comorbidities (cases)	Cardiovascular diseases: 4	Metabolic diseases: 7	Metabolic diseases: 4
	Metabolic diseases: 4	Immune and connective tissue diseases: 3	Digestive system diseases: 2
	Digestive system diseases: 5	Respiratory system diseases: 2	Respiratory system diseases: 2
	Other: 7	Others: 5	Others: 5 cases
	No diseases: 12	No diseases: 14	No diseases: 15
ASA classification	II: 15	II: 15	II: 14
	III:15	III:16	III:14

Inflammatory Indicators and Myocardial Injury Biomarkers: Measurement of serum levels of IL-1β (DLB50, R&D Systems, Minneapolis, MN, USA), IL-6, Tumor Necrosis Factor-α (TNF-α), IMA, and H-FABP.

### Enzyme-linked immunosorbent assay (ELISA)

ELISA kits were used to analyze patient serum samples obtained at predefined intervals. Human kits IMA (EH1061, Fine Biotech, Wuhan, China), H-FABP (ab108842, Abcam, Shanghai, China), IL-1β (KE00021, Proteintech, Wuhan, China), TNF-α (PHC3011, Thermo Fisher Scientific Inc, Rockford, Illinois, USA), IL-6 (88-7066-88, Thermo Fisher Scientific Inc, Rockford, Illinois, USA) and rat kits IMA (abx258296, Biolead, Beijing, China), H-FABP (ab288590, Abcam, Shanghai, China), TNF-α (ab100785, Abcam, Shanghai, China), Rat IL-6 ELISA Kit (ERA31RBX5, Thermo Fisher Scientific Inc, Rockford, Illinois, USA) kits should all be followed according to their instructions. The optical density (OD) at 450 nm was measured with the assistance of a microplate reader (Molecular Devices, Shanghai, China).

### Cell culture and processing

Ten percent fetal bovine serum was added to DMEM (11966025, Thermo Fisher Scientific Inc, Rockford, Illinois, USA) to support the growth of H9c2 rat cardiomyocytes. The cells were cultured under conditions of 37 °C, 95% humidity, and 5% CO_2_. The cell model was established using techniques previously described (22). H9c2 cells were subjected to reoxygenation for six hours (95% air, 5% CO_2_) following three hours of hypoxia (0.1% O2, 5% CO_2_% and 95% N2). The cells were randomly divided into five groups (*n* = 3): Control group (I), H/R group (II), Sevoflurane group (III), Sevoflurane + NC inhibitor group (IV) and Sevoflurane + miR-21-5p inhibitor group (V). The Control group cells were cultured under normoxic conditions (21% O_2%_ and 5% CO_2_). Except for the Control and H/R groups, other cells were treated with sevoflurane (1.5 mM) for three 15-minute intervals before the onset of hypoxia. For the miR-21-5p inhibitor group, cells were transfected with miR-21-5p inhibitor, with NC inhibitor as the control. Cardiomyocytes (1 × 10^5^ cells per well) were starved in serum-free medium for 24 h. Following the manufacturer's instructions, HiPerFect Transfection Reagent was applied for transfection (301704, QIAGEN, Hilden, German). After 48 h, the cells were placed in a modular incubator and exposed to 1% O_2_, 5% CO_2%_ and 94% N_2_ for 12 h.

### Reverse transcription quantitative polymerase chain reaction (RT-qPCR)

Total RNA was extracted using TRIzol reagent (15596026, Thermo Fisher Scientific, Waltham, MA, USA). Next, the extracted RNA was quantified using a HD-UV90 spectrophotometer (Shandong Hold Electronic Technology Co., Weifang, China) as directed in the manual. After that, 2 *μ*g of RNA underwent reverse transcription with the Vazyme DLR102 SynScript® III One-Step RT Kit (DLR102, Vazyme Biotech Co., Ltd., Nanjing, China) to create cDNA. RT-qPCR reactions were performed using a thermal cycler (Applied Biosystems, California, USA) to conduct RT-qPCR reactions. The *ΔΔ*Ct method was applied, the initial template's quantitative data was collected (23). Small RNA U6 was used as the reference gene. Each gene's forward and downstream primer sequences for RT-qPCR are provided in (Table 2).

**Table 2:** The primer sequences for RT-qPCR.

GENE	Primer sequences (5ʹ-3ʹ)	
Hsa-miR-21-5p	Forward	ACACTCCAGCTGGGTAGCTTATCAGACTGA
	Reverse	TGGTGCGTGGAGTCG
Hsa-miR-U6	Forward	CTCGCTTCGGCAGCACA
	Reverse	AACGCTTCACGAATTTGCGT
Rno-miR-21	Forward	GTACCACCTTGTCGGGTAGC
	Reverse	ATGTCAGACAGCCCATCGAC
Rno-miR-U6	Forward	CAGCACATATACTAAAATTGGAACG
	Reverse	ACGAATTTGCGTGTCATCC

### Terminal deoxynucleotidyl transferase dUTP nick-end labeling (TUNEL)

Cardiomyocytes were cultured on coverslips for four to five days and grouped based on experimental needs. After fixing the cells for 20 min in 4% paraformaldehyde solution, cells were washed three times with PBS for 5 min each. After that, cells were subjected to Proteinase K for five minutes. The TUNEL assay was performed according to the manufacturer's instructions (40307ES20-EN, Yeasen Biotechnology Co., Ltd., Shanghai, China). After completing the reaction, cells were washed three times with PBS for 5 min each and incubated in an anti-fluorescence quenching solution containing DAPI (62248, Thermo Fisher Scientific Inc, Rockford, Illinois, USA). Apoptotic cells were observed under a fluorescence microscope using a green fluorescence filter. To calculate the apoptosis rate, the number of apoptotic cells (TUNEL-positive cells) and the total number of cells were counted. The apoptosis rate was calculated as follows: apoptosis rate = (number of TUNEL-positive cells/total number of cells) × 100%.

### CCK-8 assay

Utilizing the CCK-8 assay kit (C0037, Beyotime, Shanghai, China), cell viability was evaluated. 96-well plates were seeded with 1 × 10^3^ cells per well. After washing with phosphate-buffered saline (PBS), the cells were cultured for two hours at 37 °C with 10 *μ*l CCK-8 and 90 *μ*l serum-free medium in a 95% air and 5% CO_2_ environment. To assess the rates of cell growth, the OD at 450 nm was determined using a microplate reader (Molecular Devices, Shanghai, China).

### Western blotting (WB)

The cells were lysed for 30 min on ice in RIPA buffer (20-188, Merck, Tarmstadt, Germany), shaking every five minutes. Following a 10 min centrifugation at 4 °C and 12,000 rpm on the lysates, the supernatant was collected. To ensure uniform protein loading across groups, protein concentrations were determined using a BCA protein assay kit (PA115-01, Tengen Biochemical Technology Co., Beijing, China). Proteins were transferred to a PVDF (88518, Thermo Fisher Scientific, Waltham, MA, USA) membrane after being separated via SDS-PAGE gel electrophoresis. The membrane was then blocked with 5% non-fat milk for an hour following transfer. Following that, the membrane was then treated with the following primary antibodies for the duration of the night at 4 °C for BCL-2 (1:1,000, ab194583, Abcam, Cambridge, Massachusetts, USA), for BAX (1:1,000, ab32503, Abcam, Cambridge, Massachusetts, USA), and for GAPDH (1:5,000, 4A9L6, Thermo Fisher Scientific, Waltham, MA, USA). Three times, for ten minutes each, the membrane was washed with TBST. It was then incubated for an hour at room temperature utilizing a Mouse anti-Rabbit IgG (H + L) Cross-Adsorbed Secondary Antibody, HRP (1:20,000, 31464, Thermo Fisher Scientific, Waltham, MA, USA) diluted in 5% non-fat milk. This was following three 10 min washes with TBST washes. Ultimately, the chemiluminescent substrate was used to expose the membrane in a darkroom, and ImageJ software (National Institutes of Health, USA) was used to assess the protein bands' optical density.

### Measurement of cellular MDA and SOD

Following the manufacturer's instructions, the MDA content was determined using an MDA assay kit (EEA015, Thermo Fisher Scientific, Waltham, MA, USA), and the SOD activity was assessed using a SOD assay kit (EIASODC, Thermo Fisher Scientific, Waltham, MA, USA).

### Measurement of mitochondrial reactive oxidative species (ROS)

The MitoSOX Red mitochondrial superoxide indicator (M36008, Thermo Fisher Scientific, Waltham, MA, USA) kit was used to measure the levels of ROS in mitochondria. H9c2 cells were collected, they were washed three times with PBS and incubated for 30 min at 37 °C in the dark with MitoSOX Red. Following incubation, trypsin treatment and three PBS washes were administered to the H9c2 cells, DAPI stain (Thermo Fisher Scientific Inc) was added and incubate for 5 min. Using a fluorescent microscope (Leica Microsystems, Wetzlar, Germany), the MitoSOX fluorescence intensity in the cells was found. Additionally, the fluorescence intensity was quantified using ImageJ software (National Institutes of Health, USA).

### Statistical analysis

Data analysis was performed GraphPad Prism 9 (Dotmatics, Boston, MA, USA). Data from three or more groups were analyzed using one-way analysis of variance (ANOVA), followed by *post hoc* Tukey's test. A *P*-value <0.05 was considered statistically significant. Correlation coefficients of miR-21-5p with IMA and H-FABP were analyzed by Pearson correlation. Before performing one-way ANOVA, we conducted the Shapiro–Wilk test and Kolmogorov–Smirnov test using GraphPad Prism software to assess normality, and used Levene's test to check for homogeneity of variance. The results of all tests indicated no significant deviation from normal distribution or homogeneity of variance, meeting the assumptions required for one-way ANOVA.

## Results

### Sevoflurane alleviates myocardial I/R damage and reduces postoperative complications

**Figure 1:**
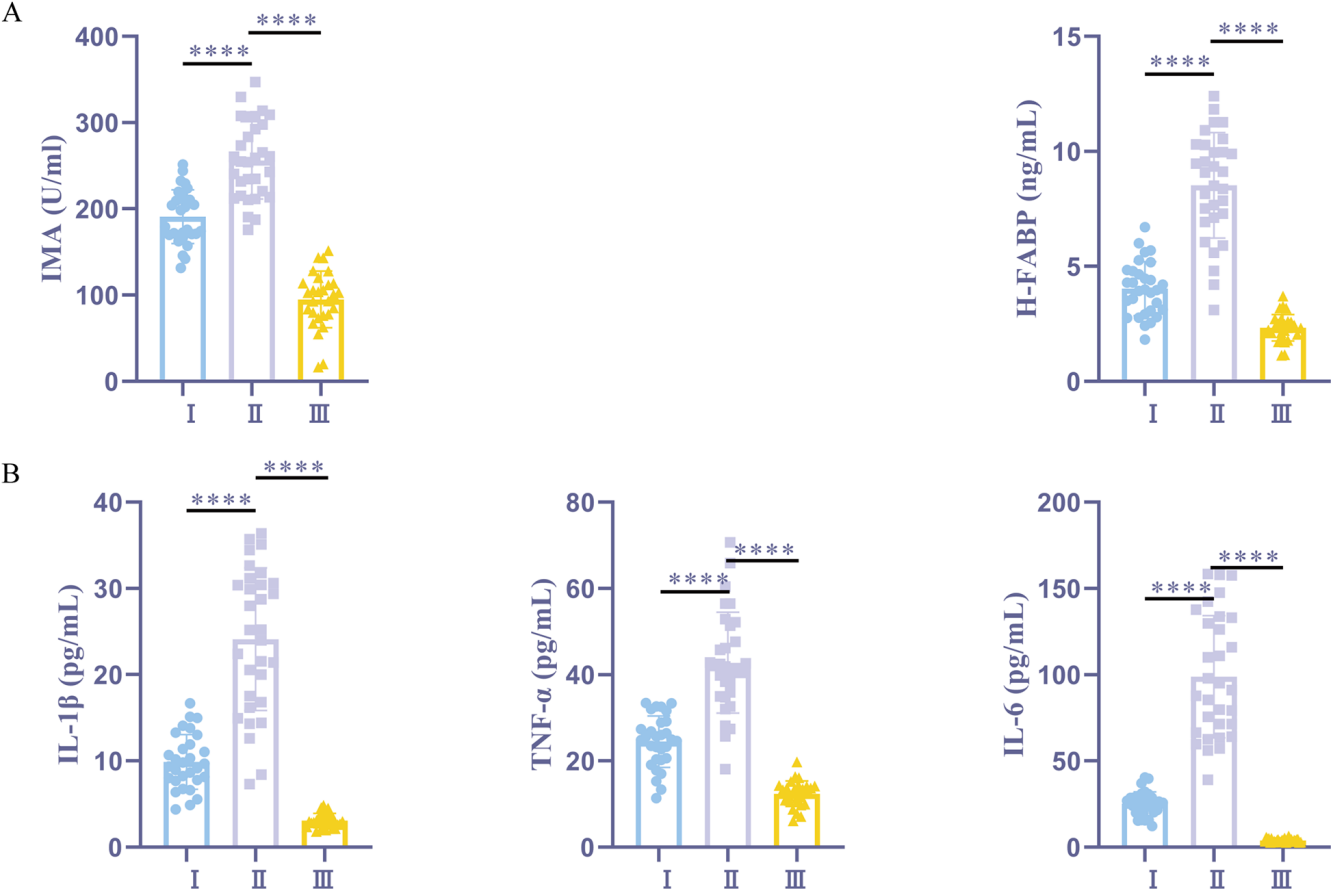
Sevoflurane alleviates myocardial I/R damage and reduces postoperative complications. **(A)** ELISA kits for IMA and H-FABP expression. **(B)** ELISA kits for IL-1β, TNF-α, and IL-6 expression. I, myocardial ischemia + sevoflurane group (*n* = 30); II, myocardial ischemia + propofol group (*n* = 31); III, normal + sevoflurane group (*n* = 28). Data were analyzed using one-way ANOVA. I vs. II, I vs. III. ****P < 0.0001.

The findings showed that, post-anesthesia, the IMA and H-FABP levels were significantly higher in the myocardial ischemia + sevoflurane group than in the normal + sevoflurane group. However, the myocardial ischemia + sevoflurane group exhibited significantly lower levels of IMA and H-FABP in comparison to the myocardial ischemia + propofol group (Figure 1A). The ELISA results showed that, compared to the normal + sevoflurane group patients, the myocardial ischemia + sevoflurane group patients had increased serum levels of the inflammatory markers. When compared to the myocardial ischemia + propofol group patients, these levels were considerably lower in the myocardial ischemia + sevoflurane group patients (Figure 1B). According to sevoflurane may help clinical patients experience fewer postoperative complications and reduced cardiac I/R damage.

### miR-21-5p is upregulated in patients with myocardial I/R damage history and is positively correlated with myocardial injury markers

**Figure 2:**
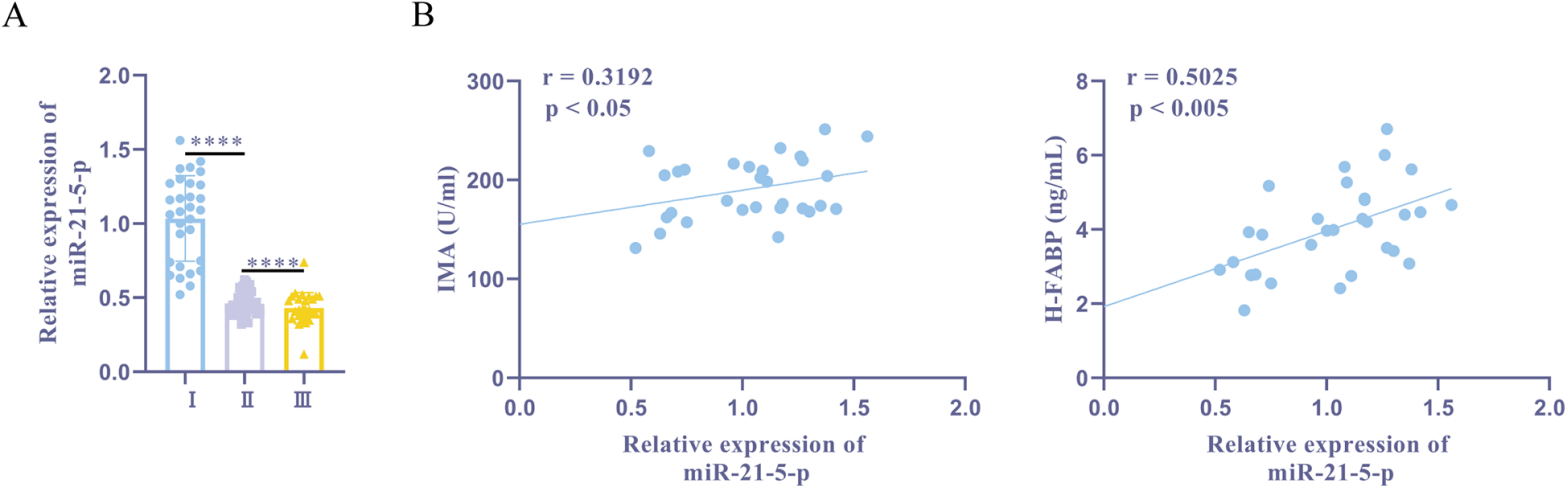
miR-21-5p is upregulated in patients with myocardial I/R damage history and is positively correlated with myocardial injury markers. **(A)** RT-qPCR detection of miR-21-5p expression. **(B)** Pearson correlation analysis of miR-21-5p with IMA and H-FABP. Ⅰ, myocardial ischemia + sevoflurane group (*n* = 30); II, myocardial ischemia + propofol group (*n* = 31); III, normal + sevoflurane group (*n* = 28). Data were analyzed using one-way ANOVA and Pearson correlation analysis. Ⅰ vs. II, Ⅰ vs. III. ****P < 0.0001.

RT-qPCR showed that, compared to the normal + sevoflurane group, the ischemia + sevoflurane group had higher expression levels of miR-21-5p. Furthermore, ischemia + sevoflurane group patients' miR-21-5p expression was significantly higher than ischemia + propofol group patients' (Figure 2A). Pearson correlation analysis in the sevoflurane group showed that miR-21-5p was positively correlated with IMA (Figure 2B). These findings show a positive association between myocardial damage indicators and an upregulation of miR-21-5p in patients with myocardial I/R damage.

### Sevoflurane preconditioning improves H/R induced cell damage and upregulates miR-21-5p

**Figure 3:**
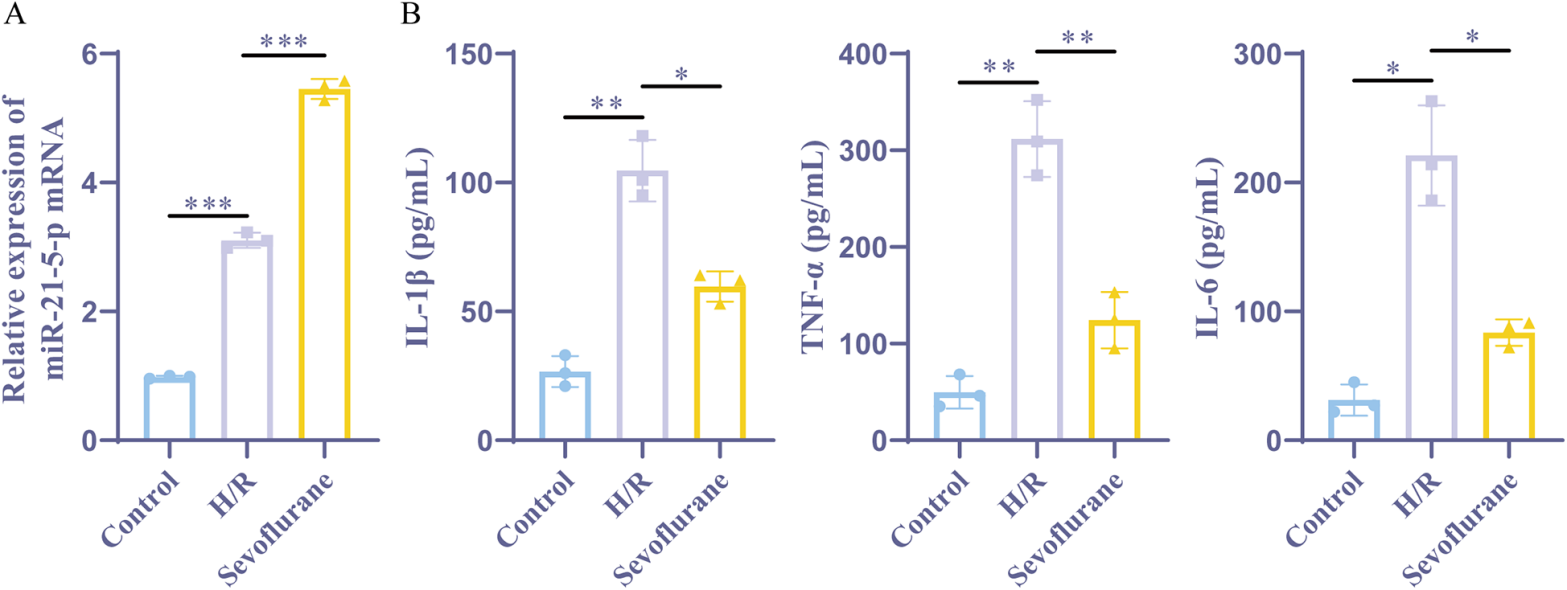
Sevoflurane preconditioning improves H/R induced cell damage and upregulates miR-21-5p. **(A)** RT-qPCR detection of miR-21-5p expression (*n* = 3). **(B)** ELISA kits for IL-1β, TNF-α, and IL-6 expression (*n* = 3). Data were analyzed using one-way ANOVA. Control vs. H/R, H/R vs. Sevoflurane. *P < 0.05, **P < 0.01, ***P < 0.001.

Next, we established a rat myocardial cell H/R model by exposing rat myocardial H9c2 cells to hypoxia for three hours and followed by reoxygenation for six hours. One hour before H/R, the sevoflurane group was treated with 2% sevoflurane. Furthermore, the results of ELISA demonstrated that the H/R group had significantly higher levels of the inflammatory markers TNF-α, IL-1β, and IL-6, than the control group. In contrast to the H/R group, the sevoflurane group showed considerably fewer amounts of these inflammatory factors and less cell damage (Figure 3B). These findings implied that an effective H/R model had been developed. The miR-21-5p was found to be considerably grown in both the H/R and sevoflurane groups when compared to the control group, according to the RT-qPCR. With a more pronounced increase in the sevoflurane group than in the H/R group (Figure 3A). These findings suggest that preconditioning with sevoflurane ameliorates H/R-induced cellular damage and encourages the increase of miR-21-5p expression.

### Sevoflurane preconditioning alleviates H/R-induced cell inflammation by upregulating miR-21-5p

**Figure 4:**
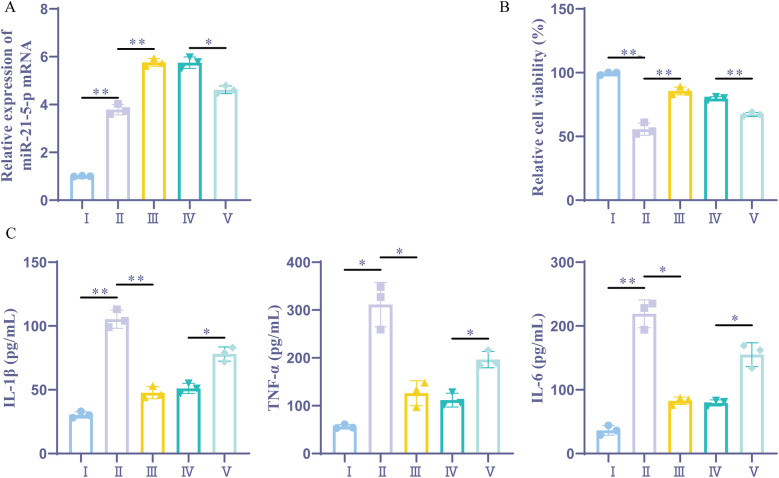
Sevoflurane preconditioning alleviates H/R-induced cell inflammation by upregulating miR-21-5p. **(A)** RT-qPCR detection of miR-21-5p expression (*n* = 3). **(B)** CCK-8 detection of cellular activity (*n* = 3). **(C)** ELISA kits for IL-1β, TNF-α, and IL-6 expression (*n* = 3). Ⅰ, control group; II, H/R group; III, sevoflurane group; IV, sevoflurane + NC inhibitor group; Ⅴ, sevoflurane + miR-21-5p inhibitor group. Data were analyzed using one-way ANOVA. Ⅰ vs. II, II vs. III, IV vs. V. *P < 0.05, **P < 0.01.

According to previous studies, miR-21-5p can reduce myocardial I/R damage (16, 19). This study aimed to investigate whether upregulating miR-21-5p through sevoflurane preconditioning could impact cardiac cell inflammation and function. This study further explored whether sevoflurane preconditioning could affect myocardial cell inflammation and activity by upregulating miR-21-5p. RT-qPCR analysis revealed, The H/R group, control group, and sevoflurane group exhibited different miR-21-5p expression levels; the expression level in the control group was lower than that in the H/R group, while the expression level in the sevoflurane group was higher than that in the H/R group, while the sevoflurane + miR-21-5p inhibitor group had a downregulated miR-21-5p compared to the sevoflurane + NC inhibitor group. CCK-8 testing results indicated that (Figure 4B), cell viability was notably decreased in the H/R group compared to the control group, whereas it was elevated in the sevoflurane group compared to the H/R group; however, the cell viability was reduced in sevoflurane + miR-21-5p inhibitor group compared to the sevoflurane + NC inhibitor group (Figure 4A). ELISA results showed that, the levels of inflammatory factors were significantly increased in the H/R group compared to the control group, while decreased in the sevoflurane group compared to the H/R group; but it was increased in the sevoflurane + miR-21-5p inhibitor group compared to the sevoflurane + NC inhibitor group (Figure 4C). These findings suggest that downregulation of miR-21-5p reverses the protective effects of sevoflurane preconditioning against H/R-induced cell damage, indicating that sevoflurane preconditioning alleviates H/R-induced cell damage by enhancing miR-21-5p expression.

### Sevoflurane preconditioning ameliorates H/R-induced cardiomyocyte apoptosis by upregulating miR-21-5p

**Figure 5:**
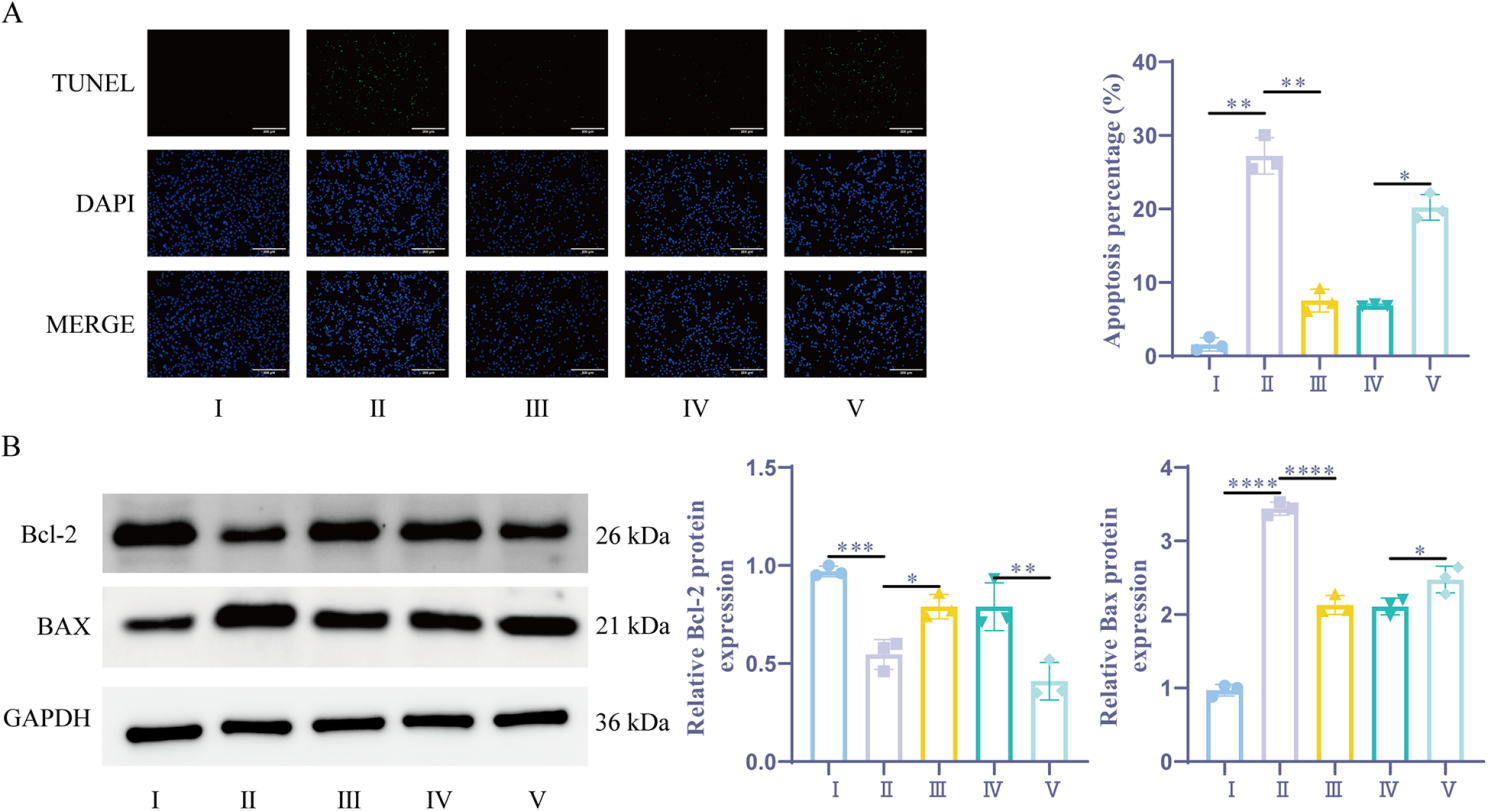
Sevoflurane preconditioning ameliorates H/R-induced cardiomyocyte apoptosis by upregulating miR-21-5p. **(A)** TUNEL detection of apoptosis (*n* = 3). **(B)** WB detection of BCL-2, BAX expression (*n* = 3). Ⅰ, control group; II, H/R group; III, sevoflurane group; IV, sevoflurane + NC inhibitor group; Ⅴ, sevoflurane + miR-21-5p inhibitor group. Data were analyzed using one-way ANOVA. Ⅰ vs. II, II vs. III, IV vs. V. *P < 0.05, **P < 0.01, ***P < 0.001, ****P < 0.0001.

TUNEL analysis revealed that, the apoptosis rate was notably greater in the H/R group and the sevoflurane group compared to the Control group. Additionally, the apoptosis rate in the sevoflurane group was considerably lower than that in the H/R group. The sevoflurane + miR-21-5p inhibitor group exhibited a considerably higher apoptotic rate compared to the sevoflurane + NC inhibitor group (Figure 5A). The WB assay results demonstrated that the level of BAX expression was dramatically elevated in the H/R group and the sevoflurane group as compared to the Control group, while the amount of BCL-2 expression was significantly reduced. Furthermore, when compared to the H/R group, the sevoflurane group's BAX expression level showed a notable reduction and BCL-2 expression was notably increased; on the other hand, compared to the sevoflurane + NC inhibitor group, the sevoflurane + miR-21-5p inhibitor group exhibited significantly increased BAX expression and decreased BCL-2 expression (Figure 5B). Results suggest that sevoflurane preconditioning can improve H/R-induced myocardial cell apoptosis by promoting the upregulation of miR-21-5p expression. Conversely, inhibition of miR-21-5p reverses the effect of sevoflurane preconditioning in ameliorating H/R-induced cell apoptosis.

### Sevoflurane preconditioning reduces H/R-induced oxidative stress by upregulating miR-21-5p

**Figure 6:**
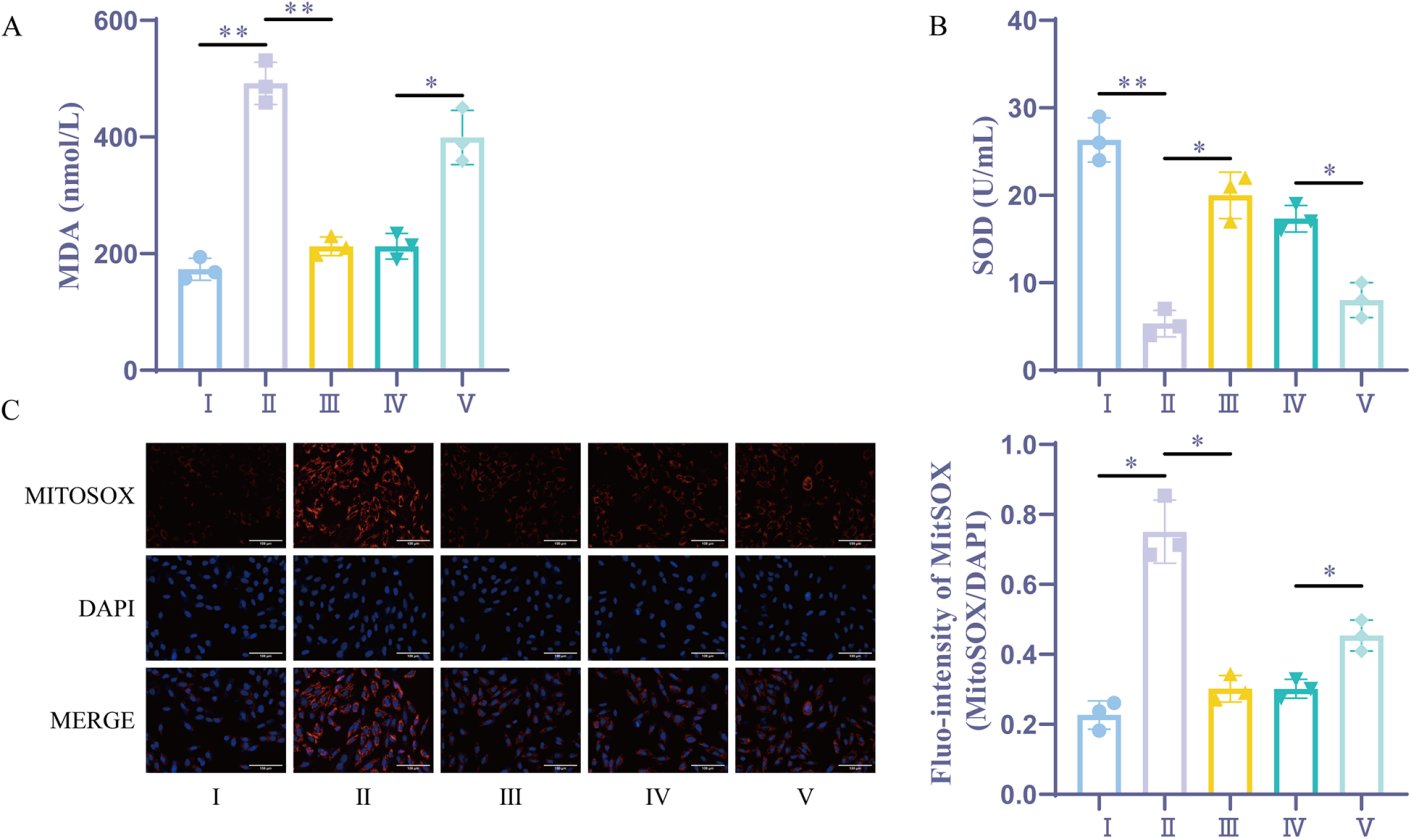
Sevoflurane preconditioning reduces H/R-induced oxidative stress by upregulating miR-21-5p. **(A)** MDA kit for the detection of MDA (*n* = 3). **(B)** SOD kit for the detection of SOD (*n* = 3). **(C)** Mitochondrial ROS measurement (*n* = 3). Ⅰ, control group; II, H/R group; III, sevoflurane group; IV, sevoflurane + NC inhibitor group; Ⅴ, sevoflurane + miR-21-5p inhibitor group. Data were analyzed using one-way ANOVA. Ⅰ vs. II, II vs. III, IV vs. V. *P < 0.05, **P < 0.01.

Through the measurement of cellular MDA and SOD levels, it was found that the H/R group and the sevoflurane group had considerably higher levels of oxidative stress and lower levels of antioxidant capacity when compared to the Control group. Additionally, the sevoflurane group showed considerably lower oxidative stress and higher antioxidant capacity compared to the H/R group. On the other hand, the sevoflurane + miR-21-5p inhibitor group had lower antioxidant capacity and elevated oxidative stress compared to the sevoflurane + NC inhibitor group (Figures 6A,B). In comparison to the Control group, the H/R group and the sevoflurane group showed a substantial rise in MitoSOX fluorescence intensity and a significant enhancement in mitochondrial oxidative stress in the MitoSOX experiment. Furthermore, the sevoflurane group demonstrated weaker mitochondrial oxidative stress and significantly lower MitoSOX fluorescence intensity compared to the H/R group (Figure 6C). However, the sevoflurane + miR-21-5p inhibitor group displayed significantly higher mitochondrial oxidative stress itor group.These findings revealed that sevoflurane preconditioning's ability to lessen H/R-induced oxidative stress is counteracted by downregulation of the miR-21-5p, indicating that sevoflurane preconditioning promotes the overexpression of miR-21-5p, which reduces oxidative damage caused by H/R.

To further investigate the mechanism by which sevoflurane alleviates cardiomyocyte apoptosis, we performed KEGG enrichment analysis on the downstream targets of miR-21-5p using Microbix (https://www.bioinformatics.com.cn/login/). The results revealed that the downstream targets of miR-21-5p were primarily enriched in the MAPK signaling pathway, which ranked second. Previous studies have reported that miR-21-5p can promote ERK-MAPK phosphorylation (24), and the activation of the ERK-MAPK signaling pathway helps reduce cardiomyocyte apoptosis in cardiac ischemia-reperfusion (I/R) preconditioning (25). Therefore, miR-21-5p may inhibit cardiomyocyte apoptosis by promoting the activation of the ERK-MAPK signaling pathway. Our RT-qPCR results showed that sevoflurane intervention suppressed the downregulation of c-Fos after modeling, while miR-21-5p inhibitor intervention significantly inhibited the expression of c-Fos. The downregulation of c-Fos indicates that ERK-MAPK activation is suppressed (26). In conclusion, these results suggest that miR-21-5p may exert its effects through the ERK-MAPK pathway, but currently, there is insufficient evidence to establish a direct causal relationship. Future studies should consider using ERK-MAPK pathway activators or inhibitors for further experiments to more clearly elucidate this mechanism.

## Discussion

Cardiovascular disease, which is prevalent and complex, includes myocardial infarction (27). However, in treating AMI, myocardial I/R damage is unavoidable. Myocardial necrosis results from varying degrees of coronary ischemia and hypoxia in the coronary arteries, which causes AMI. Among cardiovascular diseases, AMI remains a life-threatening issue (28). Achieving reperfusion within one hour of AMI can reduce myocardial damage and infarct size; but, the reperfusion process itself can cause further myocardial injury, known as myocardial I/R damage (18). Myocardial I/R damage is often accompanied by inflammatory responses and tissue damage. Its complex mechanisms present significant obstacles to disease treatment (5). To gain insight into the therapeutic mechanisms of myocardial I/R and to provide new directions for its treatment, we explored the mechanism of sevoflurane's alleviation of myocardial I/R based on miRNA-21b-5p.

Currently, propofol is frequently used as an intravenous anesthetic due to its rapid recovery characteristics; however, its most notable side effect is the suppression of the cardiovascular and respiratory systems (29). Previous studies have shown that maintaining anesthesia with sevoflurane results in more stable hemodynamics compared to propofol (30). In cardiac surgery, volatile anesthetics are more effective than intravenous anesthetics in maintaining hemodynamic stability (31). Sevoflurane, a commonly used inhalational anesthetic in clinical practice, has a lesser impact on hemodynamics, and is fast-acting and easy to control compared to other volatile anesthetics (32). Our findings showed that patients with a history of myocardial ischemia showed high expression of IMA, H-FABP, and the inflammatory factors IL-1β, TNF-α, and IL-6. However, lower levels of IMA, H-FABP, and inflammatory factors were observed in sevoflurane-anesthetized patients compared to those who received propofol anesthesia. A similar reduction in inflammatory factors after sevoflurane treatment was observed in rat cardiomyocytes. Additionally, rat cardiomyocytes treated with sevoflurane showed increased activity, reduced oxidative stress and decreased apoptosis. Therefore, sevoflurane may be a preferred preoperative anesthetic for patients with a history of myocardial ischemia.

Cardiomyocyte inflammatory response and oxidative stress were enhanced after the onset of myocardial I/R (33, 34). Following H/R, cells exhibit increased expression of inflammatory factors and heightened inflammatory responses. Oxidative stress, combined with excessive inflammation, leads to severe and irreversible damage to cardiomyocytes (35, 36). Our results showed a decrease in inflammatory factors, an increase in SOD activity, a decrease in MDA levels, and an increase in MitoSOX fluorescence intensity following sevoflurane treatment. However, we reversed the results of sevoflurane treatment reducing inflammatory factors and oxidative stress after intervention by miR-21-5p inhibitor. This suggests that sevoflurane may mitigate cellular inflammatory responses and oxidative stress by promoting miR-21-5p expression.

Apoptosis is one of the major causes of cell death in myocardial I/R (37) and is regulated by the expression levels of BAX protein and Bcl-2 protein (38). Sevoflurane pretreatment decreased the BAX expression level and increased the BCL-2 expression level, while decreasing the apoptosis rate. In addition, this study found that sevoflurane pretreatment up-regulated miR-21-5p expression. However, the addition of a miR-21-5p inhibitor reversed the sevoflurane-induced upregulation of miR-21-5p and reduced the inhibition of apoptosis. These findings suggest that sevoflurane pretreatment inhibits cardiomyocyte apoptosis through the upregulation of miR-21-5p.

Inhibition of cell apoptosis can alleviate myocardial I/R injury (39). However, how miR-21-5p alleviates myocardial cell apoptosis remains unclear. Through KEGG enrichment analysis, we found that the downstream targets of miR-21-5p are primarily enriched in the ERK-MAPK signaling pathway. Activation of the ERK-MAPK pathway can reduce myocardial cell apoptosis in cardiac ischemic preconditioning. Furthermore, miR-21-5p can promote the phosphorylation of ERK-MAPK (24). Additionally, we found that inhibition of miR-21-5p upregulation suppressed c-Fos expression. The downregulation of c-Fos indicates that ERK-MAPK phosphorylation is inhibited (40). Although these data suggest that miR-21-5p may exert its effects through the ERK-MAPK pathway, the current data only provide correlational evidence and do not establish a direct causal relationship between the two. To further validate this mechanism, future studies could use ERK-MAPK pathway activators or inhibitors, combined with more in-depth molecular biological analyses, to confirm the role of miR-21-5p in myocardial cell apoptosis.

In addition, we found that miR-21-5p exerts significant protective effects in alleviating myocardial ischemia/reperfusion (I/R) injury. Moreover, previous studies have shown that the upregulation of miR-21-5p also demonstrates cardioprotective effects in chronic heart failure (41). However, the clinical translational potential of miR-21-5p still requires further validation, as clinical individual differences, timing of treatment, and long-term efficacy evaluation may pose challenges to its application.

In summary, we have preliminarily confirmed the protective effect of sevoflurane in myocardial I/R and its regulation of miR-21-5p expression through clinical research. Additionally, this study primarily explored how sevoflurane alleviates myocardial cell injury by influencing the expression of miR-21-5p. It demonstrated the mechanism of sevoflurane's protective effect on myocardial cells by inhibiting inflammation, oxidative stress, and reducing cell apoptosis. For the first time, we have shown that sevoflurane improves myocardial cell injury in myocardial I/R by upregulating miR-21-5p. This finding suggests the potential of sevoflurane as the preferred anesthetic for myocardial ischemia patients undergoing surgery and provides a potential therapeutic target for the treatment of myocardial I/R.

## Conclusions

Sevoflurane preconditioning reduces cardiac cell damage induced by myocardial I/R injury through the upregulation of miR-21-5p. This study contributes to a better understanding of the molecular mechanisms underlying sevoflurane's myocardial protective effects and offers new insights into potential strategies for clinical treatment.

## Data Availability

The original contributions presented in the study are included in the article/Supplementary Material, further inquiries can be directed to the corresponding author.
